# Supplementary material for: Nanoradian-scale precision in light rotation measurement via indefinite quantum dynamics
Source: Sci Adv. 2024 Jul 10;10(28):eadm8524. doi: 10.1126/sciadv.adm8524 (PMC11758439; doi:10.1126/sciadv.adm8524)
Supplement: Supplementary file 1 — Notes S1 to S4 Figs. S1 to S4 Tables S1 and S2 References [file sciadv.adm8524_sm.pdf]

Science Advances
AAAS

Supplementary Materials for

Nanoradian-scale precision in light rotation measurement via indefinite quantum dynamics

Binke Xia *et al.*

Corresponding author: Jingzheng Huang, jzhuang1983@sjtu.edu.cn; Guihua Zeng, ghzeng@sjtu.edu.cn

Sci. Adv. **10**, eadm8524 (2024)
DOI: 10.1126/sciadv.adm8524

This PDF file includes:

Notes S1 to S4
Figs. S1 to S4
Tables S1 and S2
References

Supplementary Note 1: Implementation of indefinite-time-direction evolving process

In practice, it is difficult to implement the backward evolving $\hat{U}_S^\dagger$ by directly reversing the time direction. Here, we'll give a practical implementing method for the indefinite-time-direction evolving process in general. As we stated in the main text, the unknown parameter g is encoded into the Hamiltonian $\hat{H}_S$ of the parameterizing dynamics in the SQPE scheme. While a two-level auxiliary meter is employed additionally in the IQPE scheme. In most cases, we can perform a measurement operator $\hat{\sigma}_z = |0\rangle\langle 0| - |1\rangle\langle 1|$ on the auxiliary meter during the parameterizing process to implement the superposition of forward evolution and backward evolution, where the Hamiltonian of the entire system can be denoted as $\hat{H}_I = \hat{H}_S \otimes \hat{\sigma}_z$. When the parameterizing Hamiltonian $\hat{H}_S$ is time-independent, or it is time-dependent but commutative at different times, the unitary evolution of the entire system can be derived as the indefinite-time-direction form $\hat{U}_I = \hat{U}_S|0\rangle\langle 0| + \hat{U}_S^\dagger|1\rangle\langle 1|$. The derivation is given as following:

Case 1. If the Hamiltonian $\hat{H}_S$ is time-independent, then the unitary parameterizing evolution of the SQPE scheme can be denoted as $\hat{U}_S = \exp(-\mathrm{i}\hat{H}_S T)$, where T is the evolving time. As the Hamiltonian of our IQPE scheme is $\hat{H}_I = \hat{H}_S \otimes \hat{\sigma}_z$, the corresponding unitary evolution is

$$\begin{aligned}\hat{U}_I &= \exp(-\mathrm{i}\hat{H}_S \otimes \hat{\sigma}_z T) = \exp(-\mathrm{i}\hat{H}_S T \otimes \hat{\sigma}_z) \\ &= \exp(-\mathrm{i}\hat{H}_S T)|0\rangle\langle 0| + \exp(\mathrm{i}\hat{H}_S T)|1\rangle\langle 1| \\ &= \hat{U}_S|0\rangle\langle 0| + \hat{U}_S^\dagger|1\rangle\langle 1|. \end{aligned} \tag{S1}$$

Case 2. If the Hamiltonian $\hat{H}_S$ is time-dependent, but commutative at different times, the unitary evolution of the SQPE scheme can be denoted as $\hat{U}_S = \exp(-\mathrm{i}\int_0^T \hat{H}_S \mathrm{d}t)$. Similarly, the unitary evolution of our IQPE scheme can be derived as

$$\hat{U}_I = \exp\left(-\mathrm{i}\int_0^T \hat{H}_S \otimes \hat{\sigma}_z \mathrm{d}t\right) = \exp\left[-\mathrm{i}\left(\int_0^T \hat{H}_S \mathrm{d}t\right) \otimes \hat{\sigma}_z\right], \tag{S2}$$

which can be rewritten as

$$\hat{U}_I = \exp\left(-\mathrm{i}\int_0^T \hat{H}_S \mathrm{d}t\right)|0\rangle\langle 0| + \exp\left(\mathrm{i}\int_0^T \hat{H}_S \mathrm{d}t\right)|1\rangle\langle 1| = \hat{U}_S|0\rangle\langle 0| + \hat{U}_S^\dagger|1\rangle\langle 1|. \tag{S3}$$

Case 3. As for the most complicated case that the Hamiltonian $\hat{H}_S$ is time-dependent, and not commutative at different times, the unitary evolution of the SQPE scheme should be written as the Dyson series

$$\hat{U}_S = \hat{\mathbb{I}} + \sum_{n=1}^{+\infty}(-\mathrm{i})^n \int_0^T \mathrm{d}t_1 \int_0^{t_1} \mathrm{d}t_2 \cdots \int_0^{t_{n-1}} \mathrm{d}t_n \hat{H}_S(t_1)\hat{H}_S(t_2)\cdots\hat{H}_S(t_n), \tag{S4}$$

and its corresponding backward evolution is given as

$$\hat{U}_S^\dagger = \hat{\mathbb{I}} + \sum_{n=1}^{+\infty}\mathrm{i}^n \int_0^T \mathrm{d}t_1 \int_0^{t_1} \mathrm{d}t_2 \cdots \int_0^{t_{n-1}} \mathrm{d}t_n \hat{H}_S(t_n)\cdots\hat{H}_S(t_2)\hat{H}_S(t_1). \tag{S5}$$

If we still apply the Hamiltonian $\hat{H}_I = \hat{H}_S \otimes \hat{\sigma}_z$ for the indefinite-time-direction strategy, the unitary evolution of the entire system should be derived as

$$\begin{aligned}\hat{U}_I =& \hat{\mathbb{I}} + \sum_{n=1}^{+\infty}(-\mathrm{i})^n \int_0^T \mathrm{d}t_1 \int_0^{t_1} \mathrm{d}t_2 \cdots \int_0^{t_{n-1}} \mathrm{d}t_n \hat{H}_S(t_1)\hat{H}_S(t_2)\cdots\hat{H}_S(t_n) \otimes \hat{\sigma}_z^{\otimes n} \\ =& |0\rangle\langle 0| + \sum_{n=1}^{+\infty}(-\mathrm{i})^n \int_0^T \mathrm{d}t_1 \int_0^{t_1} \mathrm{d}t_2 \cdots \int_0^{t_{n-1}} \mathrm{d}t_n \hat{H}_S(t_1)\hat{H}_S(t_2)\cdots\hat{H}_S(t_n) \otimes |0\rangle\langle 0| \\ &+ |1\rangle\langle 1| + \sum_{n=1}^{+\infty}\mathrm{i}^n \int_0^T \mathrm{d}t_1 \int_0^{t_1} \mathrm{d}t_2 \cdots \int_0^{t_{n-1}} \mathrm{d}t_n \hat{H}_S(t_1)\hat{H}_S(t_2)\cdots\hat{H}_S(t_n) \otimes |1\rangle\langle 1| \\ \neq& \hat{U}_S|0\rangle\langle 0| + \hat{U}_S^\dagger|1\rangle\langle 1|, \end{aligned} \tag{S6}$$

which doesn't lead to the superposition of forward evolution and backward evolution. To implement the indefinite-time-direction evolution in this case, we should first determine the Hamiltonian $\hat{H}_S^{'}$ for the backward evolution $\hat{U}_S^{\dagger}$. Since the evolution operator $\hat{U}_S$ satisfies the Schrödinger equation $\mathrm{i}\partial_t\hat{U}_S = \hat{H}_S\hat{U}_S$, its backward evolution satisfies a similar equation $\mathrm{i}\partial_t\hat{U}_S^{\dagger} = -\hat{U}_S^{\dagger}\hat{H}_S = -\hat{U}_S^{\dagger}\hat{H}_S\hat{U}_S\hat{U}_S^{\dagger}$. Then the Hamiltonian $\hat{H}_S^{'}$ for the backward evolution for the backward evolution can be written as $\hat{H}_S^{'} = -\hat{U}_S^{\dagger}\hat{H}_S\hat{U}_S$. Theoretically, the most general method for implementing the indefinite-time-direction evolving is to devise the Hamiltonian $\hat{H}_I$ on the entire system as

$$\hat{H}_I = \hat{H}_S|0\rangle\langle 0| - \hat{U}_S^{\dagger}\hat{H}_S\hat{U}_S|1\rangle\langle 1|. \tag{S7}$$

Implementing this Hamiltonian is complicated but achievable in practice. Since the exact form of the Hamiltonian $\hat{H}_S^{'}$ for the backward evolution $\hat{U}_S^{\dagger}$ is derived, it can be achieved in a practical system with forward time direction.

Consequently, in most cases, we can apply a measurement operator $\hat{\sigma}_z = |0\rangle\langle 0| - |1\rangle\langle 1|$ on the auxiliary meter during the parameterizing process to implement the indefinite-time-direction evolution, where the Hamiltonian of the coupled system of probe and auxiliary meter is given as $\hat{H}_I = \hat{H}_S \otimes \hat{\sigma}_z$. This configuration leads to a superposition of forward evolution and backward evolution $\hat{U}_I = \hat{U}_S|0\rangle\langle 0| + \hat{U}_S^{\dagger}|1\rangle\langle 1|$ when the parameterizing Hamiltonian $\hat{H}_S$ is time-independent, or it is time-dependent but commutative at different times. For the most general case, i.e., the Hamiltonian $\hat{H}_S$ is time-dependent, and not commutative at different times, we should devise the Hamiltonian $\hat{H}_I$ on the entire system as $\hat{H}_I = \hat{H}_S|0\rangle\langle 0| - \hat{U}_S^{\dagger}\hat{H}_S\hat{U}_S|1\rangle\langle 1|$, which is more complicated but still achievable for implementation in practice.

Supplementary Note 2: Generation method of LG beams

In this work, we generate the LG beams through a SLM and a 4-f spatial filter system[44], the generation scheme is illustrated in Figure S1.

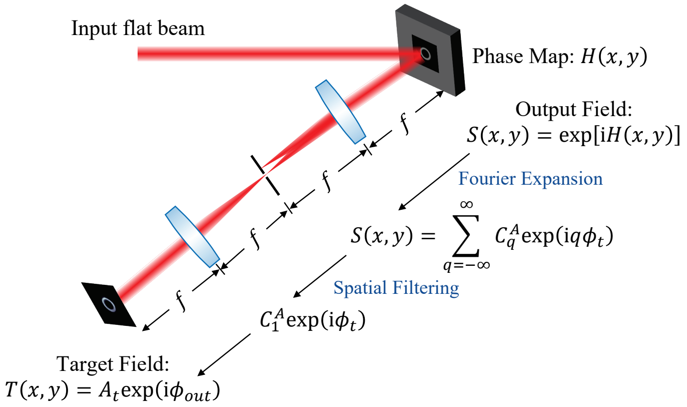

Figure S1. Generation scheme of LG beams. The expanded Gaussian beam is inputted into the spatial light modulator (SLM), and a 4-f spatial filter system is used to choose the expected mode.

In this scheme, the light beam from the laser was expanded to a approximate flat beam through a beam expander. Then inputting this beam into the SLM, where the phase map $H(x,y)$ is displayed. The output field of SLM is denoted as $S(x,y) = \exp[\mathrm{i}H(x,y)]$. Here, we denote the desired field in the target plane as $T(x,y) = A_t\exp(\mathrm{i}\phi_t)$. To filter the target light, we let

$$H(x,y) = \mathcal{M}(x,y)\cdot\Phi(x,y), \tag{S8}$$

where $\mathcal{M}$ is a normalized bounded positive function of amplitude, i.e., $0 \le \mathcal{M} \le 1$, and $\Phi \in [0,2\pi]$ is an analytical function of the amplitude and phase profile of the desired field. A calculation based on a Taylor-Fourier expansion shows that the out field of SLM can be rewritten as[45]

$$\begin{aligned} S(x,y) &= \sum_{n=-\infty}^{\infty} \frac{\sin[\pi(\mathcal{M}-n)]}{\pi(\mathcal{M}-n)} \exp[\mathrm{i}\pi(\mathcal{M}-n)]\exp(\mathrm{i}n\Phi) \\ &= \sum_{n=-\infty}^{\infty} \mathrm{sinc}(n-\mathcal{M})\exp[\mathrm{i}n\Phi + \mathrm{i}\pi(\mathcal{M}-n)], \end{aligned} \tag{S9}$$

where $\text{sinc}(\varepsilon) = (\pi\varepsilon)^{-1}\sin(\pi\varepsilon)$ is a normalized sinc function, and its property $\text{sinc}(-\varepsilon) = \text{sinc}(\varepsilon)$ is used in the above equation. By incorporating a grating phase $\phi_g(x, y)$ into the pahse term $\Phi(x, y)$, the desired filed can be filtered from the 1st-order diffraction beam[46, 47] by assigning

$$\mathcal{M}(x, y) = 1 - \text{sinc}^{-1}[A_t(x, y)], \tag{S10}$$

$$\Phi(x, y) = \text{Mod}\left[\phi_t(x, y) + \phi_g(x, y) - \pi\mathcal{M}(x, y) + \pi,\, 2\pi\right]. \tag{S11}$$

Substituting Eq. (S10) and Eq. (S11) into Eq. (S8), the phase map corresponding to the desired light field can be calculated finally. In Table S1, we list the experimental results of generated LG beams used in our experiments, OAM=1 stands for the fundamental Gaussian beam. In addition, we have estimated the fidelity of our experimental beams.

Table S1. Experimental results of generated LG beams.

OAM	0	1	4	7	10	20	30	50	80	100	150
Phase map	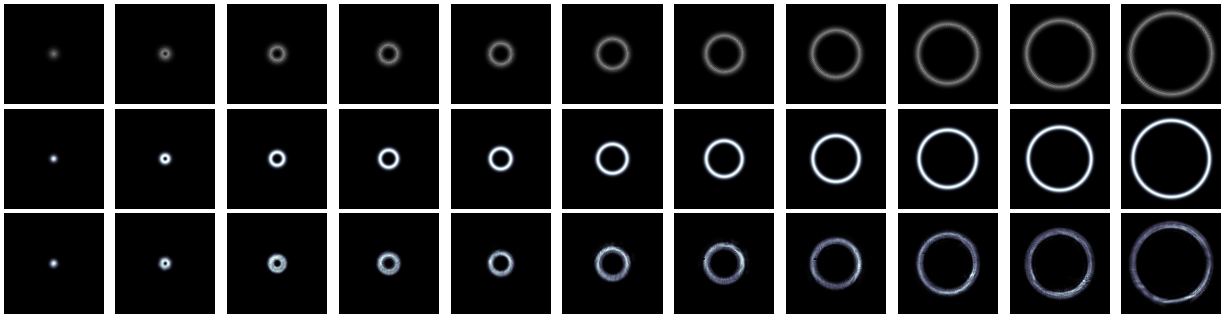										
Theory											
Experiment											
Fidelity	99.75%	99.72%	96.56%	96.52%	98.82%	95.28%	93.73%	94.87%	93.76%	90.75%	91.76%

Theoretically, the fidelity of an experimental light filed $\psi_{\text{exp}}(x, y)$ can be calculated by

$$\mathbf{F} = \frac{\left[\iint \psi_{\text{exp}}(x, y)\psi^*_{\text{th}}(x, y)\text{d}x\text{d}y\right]^2}{\iint |\psi_{\text{exp}}(x, y)|^2\text{d}x\text{d}y \iint |\psi_{\text{th}}(x, y)|^2\text{d}x\text{d}y}, \tag{S12}$$

where $\psi_{\text{th}}(x, y)$ is the desired field in theory. In practical experiment, we use the detected intensity distribution $I_{\text{exp}}(x, y) = |\psi_{\text{exp}}(x, y)|^2$ to calculate the fidelity for simplicity. Then the fidelity can be calculated approximately as:

$$\mathbf{F} \approx \frac{\left[\iint \sqrt{I_{\text{exp}}(x, y)I_{\text{th}}(x, y)}\text{d}x\text{d}y\right]^2}{\iint I_{\text{exp}}(x, y)\text{d}x\text{d}y \iint I_{\text{th}}(x, y)\text{d}x\text{d}y}. \tag{S13}$$

The results in Table S1 shows that the LG beams used in our experiments have high fidelity over 90%.

Supplementary Note 3: Configuration of practical projective measurements

To implement the practical projective measurements, the compensation of the additional relative phase $\Delta\varphi$ caused by systematic imperfections is necessary, which can be achieved by incorporating a QWP and a HWP prior to the projection of the PBS. In this section, we aim to provide a comprehensive explanation of the compensation method. As depicted in Figure S2, the optical axis of QWP is oriented at an angle of 45° with respect to the horizontal plane, while the optical axis of HWP is adjusted to an angle θ_h relative to the horizontal plane. The unitary evolution associated with the QWP and the HWP can be represented by the Jones matrices:

$$\hat{U}_{\frac{\lambda}{4}}\left(\frac{\pi}{4}\right) = \frac{1}{\sqrt{2}}\begin{pmatrix} 1 & -\text{i} \\ -\text{i} & 1 \end{pmatrix}, \qquad \hat{U}_{\frac{\lambda}{2}}(\theta_h) = \begin{pmatrix} \cos 2\theta_h & \sin 2\theta_h \\ \sin 2\theta_h & -\cos 2\theta_h \end{pmatrix}, \tag{S14}$$

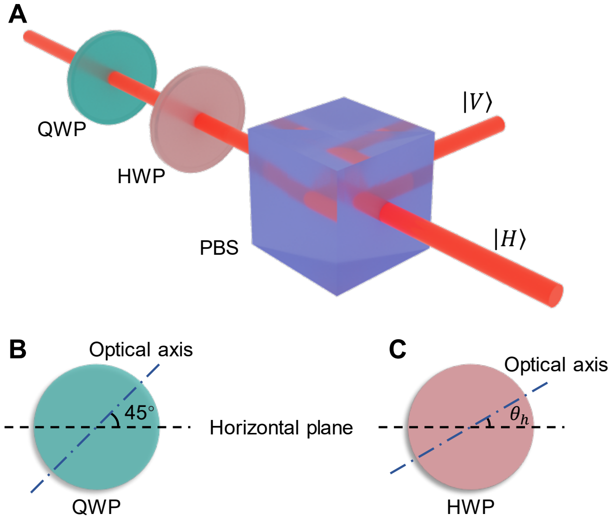

Figure S2. Configuration of the QWP and HWP before the projection of the PBS. **A** Placement sequence of the QWP, HWP and PBS for the compensated projective measurements. **B** Configuration of the QWP's optical axis. **C** Configuration of the HWP's optical axis.

respectively. As a result, the collective arrangement of the PBS, QWP, and HWP facilitates a set of orthogonal projective measurements $\{|m_1\rangle\langle m_1|, |m_2\rangle\langle m_2|\}$, where the bases

$$|m_1\rangle = \hat{U}^{\dagger}_{\frac{\lambda}{4}}\hat{U}^{\dagger}_{\frac{\lambda}{2}}|H\rangle = \frac{1}{\sqrt{2}}(\mathrm{e}^{\mathrm{i}2\theta_h}|H\rangle + \mathrm{i}\mathrm{e}^{-\mathrm{i}2\theta_h}|V\rangle), \tag{S15}$$

$$|m_2\rangle = \hat{U}^{\dagger}_{\frac{\lambda}{4}}\hat{U}^{\dagger}_{\frac{\lambda}{2}}|V\rangle = \frac{1}{\sqrt{2}}(\mathrm{e}^{\mathrm{i}2\theta_h}|H\rangle - \mathrm{i}\mathrm{e}^{-\mathrm{i}2\theta_h}|V\rangle). \tag{S16}$$

Taking into account the presence of the additional relative phase $\Delta\varphi$, the final state is revised as:

$$|\Psi_f\rangle = \frac{1}{\sqrt{2}}\left(\mathrm{e}^{-\mathrm{i}l\alpha-\mathrm{i}\frac{\Delta\varphi}{2}}|H\rangle + \mathrm{e}^{\mathrm{i}l\alpha+\mathrm{i}\frac{\Delta\varphi}{2}}|V\rangle\right)|l\rangle. \tag{S17}$$

By applying the projective measurements $\{|m_1\rangle\langle m_1|, |m_2\rangle\langle m_2|\}$ to this state, we can obtain the corresponding projective probabilities:

$$P_1 = \frac{1}{2}\left[1 + \sin\left(2l\alpha + \Delta\varphi + 4\theta_h\right)\right], \tag{S18}$$

$$P_2 = \frac{1}{2}\left[1 - \sin\left(2l\alpha - \Delta\varphi - 4\theta_h\right)\right]. \tag{S19}$$

In order to compensate for the additional relative phase $\Delta\varphi$, the orientation angle of HWP's optical axis should be adjusted as

$$\theta_h = -\frac{\Delta\varphi}{4}. \tag{S20}$$

During the experiments, the initial step involves the generation of a Gaussian beam with OAM mode $l = 0$. Subsequently, the HWP is carefully rotated to achieve a state where the received optical powers are balanced between the two photodetectors. This balanced power distribution ensures the requisite compensation for the undesired phase shift, thereby enabling accurate measurements and analysis.

Supplementary Note 4: Demodulating rotation amplitude under same frequency interference

In our experiments, we affix the PZT chips onto the reflective surface of the Dove prism as depicted in Figure S3**A**. Ideally, when we apply two sinusoidal signals with opposite phases to the top-row and bottom-row PZT chips, only an angular rotation signal is expected for the beam profile. This configuration is illustrated in Figure S3**B**.

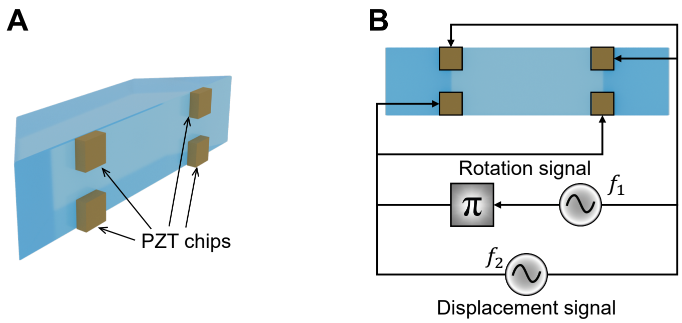

Figure S3. Configuration of PZT chips for generating angular rotation signals. **A** Arrangement of the PZT chips. **B** Generation method of the marking rotation signal and the reference displacement signal.

Nevertheless, due to the misalignment between the propagation direction of the beam and the rotational axis of the Dove prism, an unintended transverse displacement of the light beam occurs when we apply the angular rotation modulation. This displacement results in a interference on the relative phase at the same frequency as the rotation signal. To demodulate the rotation amplitude in the presence of the same-frequency interference, we introduce another sinusoidal signal with frequency f_2 to the four PZT chips. This signal induces an additional displacement signal at frequency f_2 for the beam profile. By incorporating this configuration, we create a reference signal for the relative phase interference at frequency f_1, allowing us to effectively separate and analyze the desired rotation amplitude.

In our experiments, we specifically set the rotation signal frequency as $f_1 = 20\,\mathrm{kHz}$ and the displacement signal frequency as $f_2 = 25\,\mathrm{kHz}$. Consequently, the total relative phase consists of three frequency components: $\Phi = \Phi^{(\mathrm{DC})} + \Phi^{(20\,\mathrm{kHz})} + \Phi^{(25\,\mathrm{kHz})}$. Here, $\Phi^{(\mathrm{DC})} = 2l\alpha^{(\mathrm{DC})} + \Delta\varphi^{(\mathrm{DC})}$ represents the zero-frequency component, which comprises the preset rotation of the beam profile and the additional relative phase stemming from systematic imperfections. This zero-frequency component can be compensated for using the method discussed in the previous section. The rotation

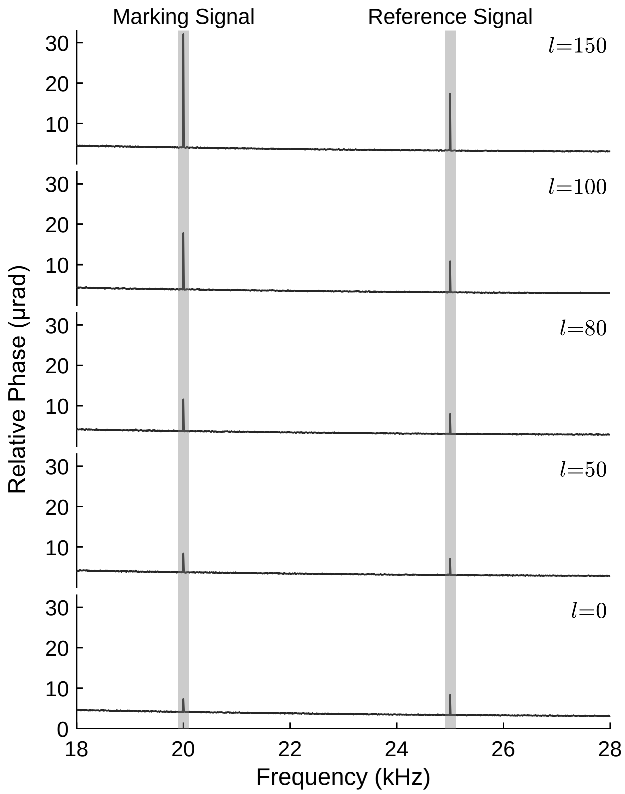

Figure S4. Amplitude spectrum of detected relative phase Φ regarding to inputting Gaussian beam ($l = 0$) and LG beams with topology charges of $l = 50, 80, 100, 150$.

signal induces a relative phase at the frequency of 20 kHz, given by $\Phi^{(20\,\mathrm{kHz})} = 2l\alpha^{(20\,\mathrm{kHz})} + \Delta\varphi^{(20\,\mathrm{kHz})}$. Here, $\alpha^{(20\,\mathrm{kHz})}$ represents the rotation amplitude of the beam profile, and $\Delta\varphi^{(20\,\mathrm{kHz})}$ corresponds to the additional relative phase induced by the unintended transverse displacement of the light beam. On the other hand, the displacement signal solely induces a reference relative phase at the frequency of 25 kHz, denoted as $\Phi^{(25\,\mathrm{kHz})} = \Delta\varphi^{(25\,\mathrm{kHz})}$. In Figure S4, we illustrate the experimental results of amplitude spectrum for total relative phase Φ at the frequency range of 18 kHz to 28 kHz. The five spectra from the bottom to top corresponds to the beams with topological charges of $l = 0, 50, 80, 100, 150$, respectively. Here, we highlight the marking signal induced relative phase $\Phi^{(20\,\mathrm{kHz})}$ and reference signal induced relative phase $\Phi^{(25\,\mathrm{kHz})}$ using gray shadows in Figure S4.

To estimate the same-frequency interference $\Delta\varphi^{(20\,\mathrm{kHz})}$ when employing light beams with different topological charges, we initially utilize a Gaussian beam as a reference. In the case of the Gaussian beam, the relative phase at the frequency of 20 kHz is solely caused by the unintended transverse displacement of the light beam ($l = 0$), then the interference relative phase $\Delta\varphi_G^{(20\,\mathrm{kHz})}$ is measured. Additionally, we measure the reference relative phase $\Delta\varphi_G^{(25\,\mathrm{kHz})}$ induced by the displacement signal. Subsequently, we generate the LG beam with a specific topological charge l to perform the demodulation of the rotation signal. The corresponding relative phase $\Phi_l^{(20\,\mathrm{kHz})}$ at the frequency of 20 kHz and $\Phi_l^{(25\,\mathrm{kHz})}$ at the frequency of 25 kHz are measured. Here, $\Phi_l^{(25\,\mathrm{kHz})} = \Delta\varphi_l^{(25\,\mathrm{kHz})}$ represents the reference signal associated with the LG beam and is employed to calculate the interference term. Specifically, the interference at 20 kHz is obtained as $\Delta\varphi_l^{(20\,\mathrm{kHz})} = \Delta\varphi_l^{(25\,\mathrm{kHz})} \cdot \Delta\varphi_G^{(20\,\mathrm{kHz})}/\Delta\varphi_G^{(25\,\mathrm{kHz})}$. Consequently, the rotation amplitude of the beam profile at the frequency of 20 kHz can be determined as

$$\alpha^{(20\,\mathrm{kHz})} = \frac{1}{2l}\left[\Phi_l^{(20\,\mathrm{kHz})} - \Phi_l^{(25\,\mathrm{kHz})} \cdot \frac{\Phi_G^{(20\,\mathrm{kHz})}}{\Phi_G^{(25\,\mathrm{kHz})}}\right]. \tag{S21}$$

In Table S2, we illustrate the experimental results of marking signal induced phase $\Phi^{(20\,\mathrm{kHz})}$ and reference signal induced phase $\Phi^{(25\,\mathrm{kHz})}$, and calculate the demodulated rotation amplitudes of the beam profile at the frequency of 20 kHz using Eq. (S21) when inputting LG beams with topological charges of 50, 80, 100 and 150, respectively. The results show that the rotation angle is around 60 nrad, which aligns well with the estimating value when we apply the rotation signal with a peak-to-peak level of 12 mV to the PZT chips.

Table S2. Demodulated rotation angles of beam profile.

	Experimental results		Demodulated results	
Inputting beams	$\Phi^{(20\,\mathrm{kHz})}$	$\Phi^{(25\,\mathrm{kHz})}$	α	SIR
Gaussian	7.34 µrad	8.36 µrad	\	\
LG_{50}	8.39 µrad	7.09 µrad	58.72 nrad	0.94
LG_{80}	11.59 µrad	7.99 µrad	52.02 nrad	1.19
LG_{100}	17.84 µrad	10.83 µrad	60.84 nrad	1.28
LG_{150}	32.12 µrad	17.39 µrad	69.67 nrad	1.37

In addition, we have estimated the signal-to-interference ratio (SIR) when demodulating the rotation amplitude of beam profile, which is calculated through

$$\mathrm{SIR} = \frac{\Phi_l^{(20\,\mathrm{kHz})} - \Delta\varphi_l^{(20\,\mathrm{kHz})}}{\Delta\varphi_l^{(20\,\mathrm{kHz})}} = \frac{\Phi_l^{(20\,\mathrm{kHz})} \cdot \Phi_G^{(25\,\mathrm{kHz})} - \Phi_l^{(25\,\mathrm{kHz})} \cdot \Phi_G^{(20\,\mathrm{kHz})}}{\Phi_l^{(25\,\mathrm{kHz})} \cdot \Phi_G^{(20\,\mathrm{kHz})}}. \tag{S22}$$

Theoretically, the SIR can be also expressed as

$$\mathrm{SIR} = 2l\frac{\alpha^{(20\,\mathrm{kHz})}}{\Delta\varphi_l^{(20\,\mathrm{kHz})}}, \tag{S23}$$

which means that the SIR can be improved by increasing the OAM value l of inputting beam when the interference $\Delta\varphi_l^{(20\,\mathrm{kHz})}$ is constant. Though $\Delta\varphi_l^{(20\,\mathrm{kHz})}$ varies with inputting beams in our experiments, we can still observe the improvement of SIR when increasing the OAM value l of inputting beam from Table S2.

REFERENCES AND NOTES

1. M. Padgett, R. Bowman, Tweezers with a twist. *Nat. Photonics* **5**, 343–348 (2011).

2. U. G. Būtaitė, G. M. Gibson, Y.-L. D. Ho, M. Taverne, J. M. Taylor, D. B. Phillips, Indirect optical trapping using light driven micro-rotors for reconfigurable hydrodynamic manipulation. *Nat. Commun.* **10**, 1215 (2019).

3. A. B. Stilgoe, T. A. Nieminen, H. Rubinsztein-Dunlop, Controlled transfer of transverse orbital angular momentum to optically trapped birefringent microparticles. *Nat. Photonics* **16**, 346–351 (2022).

4. Y. Hu, J. J. Kingsley-Smith, M. Nikkhou, J. A. Sabin, F. J. Rodríguez-Fortuño, X. Xu, J. Millen, Structured transverse orbital angular momentum probed by a levitated optomechanical sensor. *Nat. Commun.* **14**, 2638 (2023).

5. J. Courtial, D. A. Robertson, K. Dholakia, L. Allen, M. J. Padgett, Rotational frequency shift of a light beam. *Phys. Rev. Lett.* **81**, 4828–4830 (1998).

6. M. P. J. Lavery, F. C. Speirits, S. M. Barnett, M. J. Padgett, Detection of a spinning object using light's orbital angular momentum. *Science* **341**, 537–540 (2013).

7. Z. Zhang, L. Cen, J. Zhang, J. Hu, F. Wang, Y. Zhao, Rotation velocity detection with orbital angular momentum light spot completely deviated out of the rotation center. *Opt. Express* **28**, 6859–6867 (2020).

8. S. Franke-Arnold, G. Gibson, R. W. Boyd, M. J. Padgett, Rotary photon drag enhanced by a slow-light medium. *Science* **333**, 65–67 (2011).

9. Y. Gorodetski, K. Y. Bliokh, B. Stein, C. Genet, N. Shitrit, V. Kleiner, E. Hasman, T. W. Ebbesen, Weak measurements of light chirality with a plasmonic slit. *Phys. Rev. Lett.* **109**, 013901 (2012).

10. Z. Liang, D. Fan, Visible light-gated reconfigurable rotary actuation of electric nanomotors. *Sci. Adv.* **4**, eaau0981 (2018).

11. F. Pampaloni, J. Enderlein, *Gaussian, Hermite-Gaussian, and Laguerre-Gaussian beams: A primer*. arXiv: physics/0410021 (2004).

12. A. K. Jha, G. S. Agarwal, R. W. Boyd, Supersensitive measurement of angular displacements using entangled photons. *Phys. Rev. A* **83**, 053829 (2011).

13. R. Fickler, R. Lapkiewicz, W. N. Plick, M. Krenn, C. Schaeff, S. Ramelow, A. Zeilinger, Quantum entanglement of high angular momenta. *Science* **338**, 640–643 (2012).

14. F. Bouchard, P. de la Hoz, G. Björk, R. W. Boyd, M. Grassl, Z. Hradil, E. Karimi, A. B. Klimov, G. Leuchs, J. Řeháček, L. L. Sánchez-Soto, Quantum metrology at the limit with extremal majorana constellations. *Optica* **4**, 1429–1432 (2017).

15. V. D'Ambrosio, N. Spagnolo, L. Del Re, S. Slussarenko, Y. Li, L. C. Kwek, L. Marrucci, S. P. Walborn, L. Aolita, F. Sciarrino, Photonic polarization gears for ultra-sensitive angular measurements. *Nat. Commun.* **4**, 2432 (2013).

16. V. Cimini, E. Polino, F. Belliardo, F. Hoch, B. Piccirillo, N. Spagnolo, V. Giovannetti, F. Sciarrino, Experimental metrology beyond the standard quantum limit for a wide resources range. *npj Quant Inform* **9**, 20 (2023).

17. B. Xia, J. Huang, H. Li, M. Liu, T. Xiao, C. Fang, G. Zeng, Ultrasensitive measurement of angular rotations via a Hermite-Gaussian pointer. *Photonics Res.* **10**, 2816–2827 (2022).

18. C. L. Degen, F. Reinhard, P. Cappellaro, Quantum sensing. *Rev. Mod. Phys.* **89**, 035002 (2017).

19. J. Liu, H. Yuan, X.-M. Lu, X. Wang, Quantum fisher information matrix and multiparameter estimation. *J. Phys. A Math. Theor.* **53**, 023001 (2019).

20. G. Chiribella, Z. Liu, Quantum operations with indefinite time direction. *Commun. Phys.* **5**, 190 (2022).

21. Y. Guo, Z. Liu, H. Tang, X.-M. Hu, B.-H. Liu, Y.-F. Huang, C.-F. Li, G.-C. Guo, G. Chiribella, Experimental demonstration of input-output indefiniteness in a single quantum device. *Phys. Rev. Lett.* **132**, 160201 (2024).

22. T. Strömberg, P. Schiansky, M. T. Quintino, M. Antesberger, L. Rozema, I. Agresti, Č. Brukner, P. Walther, Experimental superposition of time directions. *Phys. Rev. Res.* **6**, 023071 (2022).

23. C. Helstrom, Minimum mean-squared error of estimates in quantum statistics. *Phys. Lett. A* **25**, 101–102 (1967).

24. C. Helstrom, The minimum variance of estimates in quantum signal detection. *IEEE Trans. Inf. Theory* **14**, 234–242 (1968).

25. C. W. Helstrom, Cramér-rao inequalities for operator-valued measures in quantum mechanics. *Int. J. Theor. Phys.* **8**, 361–376 (1973).

26. R. Demkowicz-Dobrzanski, M. Jarzyna, J. Kolodynski, Quantum limits in optical interferometry, *Prog. Opt.* 60, pp. 345–435 (2015).

27. *Quantum Detection and Estimation Theory*, C. W. Helstrom, ed. (Elsevier, 1976), vol. **123** of *Mathematics in Science and Engineering*, pp. 235–293.

28. R. Demkowicz-Dobrzański, W. Górecki, M. Guţă, Multi-parameter estimation beyond quantum fisher information. *J. Phys. A Math. Theor.* **53**, 363001 (2020).

29. S. Pang, A. N. Jordan, Optimal adaptive control for quantum metrology with time-dependent hamiltonians. *Nat. Commun.* **8**, 14695 (2017).

30. V. Giovannetti, S. Lloyd, L. Maccone, Quantum metrology. *Phys. Rev. Lett.* **96**, 010401 (2006).

31. Y. Israel, S. Rosen, Y. Silberberg, Supersensitive polarization microscopy using noon states of light. *Phys. Rev. Lett.* **112**, 103604 (2014).

32. R. Barboza, A. Babazadeh, L. Marrucci, F. Cardano, C. de Lisio, V. D'Ambrosio, Ultra-sensitive measurement of transverse displacements with linear photonic gears. *Nat. Commun.* **13**, 1080 (2022).

33. L. Zhang, A. Datta, I. A. Walmsley, Precision metrology using weak measurements. *Phys. Rev. Lett.* **114**, 210801 (2015).

34. G. Chen, N. Aharon, Y. N. Sun, Z. H. Zhang, W. H. Zhang, D. Y. He, J. S. Tang, X. Y. Xu, Y. Kedem, C. F. Li, G. C. Guo, Heisenberg-scaling measurement of the single-photon kerr non-linearity using mixed states. *Nat. Commun.* **9**, 93 (2018).

35. J. Schwinger, Brownian motion of a quantum oscillator. *J. Math. Phys.* **2**, 407–432 (2004).

36. J. J. Sakurai, J. Napolitano, *Modern Quantum Mechanics* (Cambridge University Press, 2018), second edn.

37. M. J. Padgett, J. Courtial, Poincaré-sphere equivalent for light beams containing orbital angular momentum. *Opt. Lett.* **24**, 430–432 (1999).

38. G. F. Calvo, Wigner representation and geometric transformations of optical orbital angular momentum spatial modes. *Opt. Lett.* **30**, 1207–1209 (2005).

39. A. Forbes, M. de Oliveira, M. R. Dennis, Structured light. *Nat. Photonics* **15**, 253–262 (2021).

40. R. Schirhagl, K. Chang, M. Loretz, C. L. Degen, Nitrogen-vacancy centers in diamond: Nanoscale sensors for physics and biology. *Annu. Rev. Phys. Chem.* **65**, 83–105 (2014). PMID: 24274702.

41. T. Zhang, G. Pramanik, K. Zhang, M. Gulka, L. Wang, J. Jing, F. Xu, Z. Li, Q. Wei, P. Cigler, Z. Chu, Toward quantitative bio-sensing with nitrogen-vacancy center in diamond. *ACS Sens.* **6**, 2077–2107 (2021).

42. Z. Xu, C. Liu, S. Zhao, S. Chen, Y. Zhao, Molecular sensors for NMR-based detection. *Chem. Rev.* **119**, 195–230 (2019).

43. R. D. Allert, K. D. Briegel, D. B. Bucher, Advances in nano- and microscale nmr spectroscopy using diamond quantum sensors. *Chem. Commun.* **58**, 8165–8181 (2022).

44. T. W. Clark, R. F. Offer, S. Franke-Arnold, A. S. Arnold, N. Radwell, Comparison of beam generation techniques using a phase only spatial light modulator. *Opt. Express* **24**, 6249–6264 (2016).

45. J. A. Davis, D. M. Cottrell, J. Campos, M. J. Yzuel, I. Moreno, Encoding amplitude information onto phase-only filters. *Appl. Optics* **38**, 5004–5013 (1999).

46. V. Arrizón, U. Ruiz, R. Carrada, L. A. González, Pixelated phase computer holograms for the accurate encoding of scalar complex fields. *J. Opt. Soc. Am. A* **24**, 3500–3507 (2007).

47. E. Bolduc, N. Bent, E. Santamato, E. Karimi, R. W. Boyd, Exact solution to simultaneous intensity and phase encryption with a single phase-only hologram. *Opt. Lett.* **38**, 3546–3549 (2013).
